# Financial development and environmental quality: Does the financial environmental Kuznets Curve Prevail in Australia?

**DOI:** 10.1016/j.heliyon.2024.e38454

**Published:** 2024-09-26

**Authors:** Ambepitiya Wijethunga Gamage Champa Nilanthi Wijethunga, Mohammad Mafizur Rahman, Tapan Sarker

**Affiliations:** a School of Business, University of Southern Queensland, West Street, Toowoomba, QLD, 4350, Australia; b Department of Accountancy & Finance, Faculty of Management Studies, Sabaragamuwa University of Sri Lanka, Belihuloya, 70140, Sri Lanka; c School of Business, University of Southern Queensland, Springfield Education City, 37 Sinnathamby Blvd, Springfield Central, QLD, 4300, Australia

**Keywords:** Financial development, Environmental Kuznets Curve (EKC), Environmental quality, Financial Environmental Kuznets Curve (FEKC), Climate change

## Abstract

It is crucial to evaluate the link between financial development and environmental quality in order to meet the environmental sustainability goals. Therefore, this scholarly work seeks to validate the of an inverted U-shaped relationship between financial development and environmental quality in Australia, which modifies the conventional theory of the Environmental Kuznets Curve. For the purposes of empirical analysis, we utilized the Autoregressive Distributed Lag bound test over the period from 1980 to 2021 for empirical investigation. Our findings confirm the existence of a Financial Environmental Kuznets Curve (FEKC) in the long-run, while the short-run results do not support its establishment. This means that achieving a financial development level of 0.0458 could help attain the environmental wellbeing in Australia. Similarly, the estimation outcomes affirm that the conventional Environmental Kuznets Curve exists in the long-run but not in the short-run. Our results also indicate that energy usage negatively impacts environmental quality, while foreign direct investments support the pollution halo effect in the long-run but do not exhibit this effect in the short-run. The roles of urbanization and trade openness are positive in enhancing quality of environment in the short-run. However, the effect of the carbon tax on determining environmental quality in Australia is deemed insignificant. In conclusion, this study offers vital policy recommendations to help achieve the Australian government's commitment to climate change.

## 1 Introduction

In recent years, growing concerns have arisen regarding the imperative need to address climate variability, rising global temperatures, and the biodiversity loss because of the detrimental pressure of human and economic activities on biosystems [1,2]. As such, today, environmental safeguard stands at the forefront of policymakers' agendas worldwide. Global organizations have undertaken a diverse range of collaborative commitments, including the Paris Agreement and the Kyoto Protocol to assure that global warming remains below the defined target levels within stipulated periods [[2], [3], [4]]. However, these initiatives would not be achievable without assistance from the financial system of the economy because financial support is essential for transforming economies into carbon-neutral economies [2]. Therefore, the modern economy requires an economic system that supported by a stable and sustainable financial system that is sensible with the environment by allocating financial resources to environmentally sustainable economic activities [5].

Primarily, the financial system is required to flow financial resources from surplus units to deficit units, facilitate payment systems, and provide other financial services [2]. Additionally, it is expected that the financial system progress parallel with the economic progress which ultimately drives economic development [2]. Meanwhile, the financial development-economic growth-environmental quality relationship has recently become an emerging research topic for many scholars because of its need and significance in understanding the route for global warming. The progress in the financial system may impact our environment via various ways: scale effect, income effect, regulation effect, and technology effect. In particular, the linear relationship among financial development (hereafter FD) and EQ (hereafter EQ) has been widely explored, yet the conclusion about the interlink between FD and EQ remains inconclusive.

The empirical takeaway of the linear relationship between FD and EQ has led to different interpretations of the relationship among variables. The first group of empirical discussions suggests that FD can affect EQ either positively or negatively [see e.g. [[6], [7], [8], [9], [10], [11], [12], [13], [14], [15], [16], [17], [18], [19], [20], [21]]. The next group of discussions highlights the insignificant role of FD in influencing EQ [see e.g. [[22], [23], [24], [25], [26], [27], [28]]. However, current empirical disclosure on FD and EQ goes beyond the linear relationship, and currently, scholars are increasingly inclined to explore the nonlinear FD-EQ link by diverging the traditional Environmental Kuznets Curve (EKC) which is now being referred to as the Financial Environmental Kuznets Curve (FEKC). The FEKC explains that at the early phase of FD, there is less attention paid to its impact on the ecosystem and as the financial system progresses beyond the threshold point, EQ improves by adopting green practices [2,29].

In Australia, as the third-largest contributor to the economy, the financial sector holds 7.6 % of the industry share of output,^1^ confirming its vital role in FD by directing savings towards productive investment opportunities. Additionally, Australia is ranked 14^th^ in the world for greenhouse gas emissions, contributing approximately 1 % to global emissions.^2^ To overcome the climate challenges, Australia has set environmental targets through enacting the Climate Change Bill 2022, which must be achieved within specified timeframes. This reinforces the nation's dedication to decreasing greenhouse gas emissions by 43 % from the 2005 benchmark by 2030, With an ultimate target of reaching net-zero emissions by 2050. It emphasizes that to reach the environmental targets of the Australian economy, the financial sector, as a leading industry of the economy, should be sensible with the EQ in Australia by allocating capital to environmentally sustainable investments rather than profit-driven investments. However, the current understanding of EQ in Australia primarily emphasizes the effect of energy usage, economic growth, urbanization, foreign direct investments, and education in determining the degree of EQ [see e.g. [[30], [31], [32], [33], [34], [35], [36], [37]]. In existing evidence, there is limited effort devoted to linking FD to EQ, which is crucial for enhancing the credibility of our comprehension of the impact of FD on EQ for informed decision-making.

Given the financial system's significance in aiding the transition to a carbon-neutral economy in Australia, this research primarily targets to validate the presence of the Financial Environmental Kuznets Curve (FEKC) for understanding the link between FD and EQ. Studying the FEKC contributes to economic theory by extending the traditional Environmental Kuznets Curve (EKC) to include the role of FD in shaping EQ. This integration provides a comprehensive understanding of development dynamics. Similarly, this study enriches the broader economic literature by linking multidimensional aspects of FD & EQ. Moreover, studying the inverted U-shaped relationship between FD and EQ is significant for informing environmental regulations. It helps policymakers balance FD and environmental sustainability by identifying specific stages where FD may harm the environment, thereby guiding targeted interventions. The novelty and uniqueness of this study is that its exploration of the existence of the FEKC in Australia as a country-specific analysis because the current consensus of an inverted U-shaped relationship between FD and EQ in Australia remains inconclusive due to the absence of country-specific studies in Australia. Additionally, this study focuses on addressing the overall aspects of FD, including the development of financial markets and financial institutions. The existing empirical studies usually model only a few selected aspects of FD, such as equity market capitalization, broad money supply, the International Monetary Fund's FD index, and domestic credit that measures financial access, depth, and efficiency, etc. Therefore, to a fill the existing gap, this study takes a integrated approach to include all components of the FD: financial access, financial depth, financial efficiency, and financial stability of financial markets and financial institutions, to measure their impact on EQ. Moreover, this study uses comprehensive measures to assess EQ. Unlike much of the existing evidence, which primarily relies on carbon emissions (CO_2_). This study considers wider measure: greenhouse gas emissions (hereafter GHG). By addressing a large range of EQ indicator and using updated data, it provides a more comprehensive perspective on FD-EQ nexus.

After the introduction, the rest of the paper is structured as below: Section 2 explores the theoretical foundations and recent empirical evidence of the FEKC. Section 3 explains methodological details, including data collection and model development, to support the study's objectives. Section 4 reviews and discusses the empirical findings. To wrap-up, Section 5 presents the study's conclusions and policy implications.

## 2 Literature review with theoretical notion

The Kuznets curve took a new pathway in 1995, as it became the fundamental concept for discussing the economic-environmental nexus. Importantly [38], adopted the Kuznets curve to study the economic-environmental relationship and revealed an inverted U-shaped association between economic progress and sulfur dioxide, which is referred to as the Environmental Kuznets Curve (EKC). As per the EKC framework, initially, the economy transfers from low economic growth to high economic growth, challenging EQ due to variations in the economic structure which produces more emissions. However, once the economy reaches a threshold level, EQ starts to enhance as the economy moves towards information and service-based economic activities and cleaner technologies. A significant amount of existing scholarly work is devoted to empirically testing the EKC and has validated its existence in diverse economic settings. For e.g. [[39], [40], [41], [42], [43]]. Later on, the finance-environmental relationship is theoretically backed by the EKC framework. More importantly [2], defined the inverted U-shaped relationship between FD and EQ as the FEKC. It describes that initially, as the financial system advances, EQ degrades. Subsequently, as the financial system matures, EQ begins to improve because low financing costs facilitate greener technology innovations and the usage of renewable energy, which decreases GHG [2,44].

The financial sector tends to pay less attention to EQ during the initial phase of economic growth [44]. However, as the economy matures, a developed financial sector begins to prioritize EQ by allocating capital to environmentally sustainable economic activities [44]. [44] confirmed the appearance of an inverted U-shaped connection between FD and CO_2_. However, the proxy variable for FD in [44] was limited to bank-based variable. Following the argument made by [[44], [45]] devoted their study to exploring the inverted U-shaped FD-EQ relationship in the United Arab Emirates. The study's econometric results empirically demonstrated the applicability of the EKC framework to the FD-EQ relationship and revealed an inverted U-shaped association between FD and CO_2_ in the UAE. Furthermore, the study measured the FD using one financial depth proxy. Similarly [46], confirmed the inverted U-shaped relationship between FD and CO_2_ in the UAE. Further, in an advancement of the EKC framework, [46] explored the N-shaped FEKC by introducing the cubic term of FD into the model because if future economic progress is driven by FD as a instrument for attaining sustainable economic growth, then FD may be positively connected with environmental degradation. Additionally, [46] employed a comprehensive selection of measures for FD, including both the equity market development and banking sector development proxies which assess the financial depth and efficiency of the banking sector and only the financial depth of the stock market. Significantly, the study addressed certain aspects of FD that were silent in the study of [45] while failing to provide a comprehensive view of FD comprising financial stability proxies in both financial markets and institutions.

The presence of FEKC is confirmed by [47], which adopted the methodology of [45]. However, Chunyu et al.'s [47] empirical work also limited FD to total domestic credit in their study and confirmed an inverted U-shaped link with carbon discharges within the study context. By expanding the measures of FD, [48] tested and established an inverted U-shaped relationship between FD and CO_2_ in 26 economies in the Asia-Pacific region. [48] study addressed certain attributes of financial market development and financial institutional development, but it failed to capture the stability of financial markets and institutions as a significant aspect of FD.

Identically, [49] examined the nonlinear FD-ecological footprint nexus across a worldwide sample. Importantly, Ashraf et al.'s [49] study employed nine measures of FD, including overall financial system development, financial market development, financial institution development, and sub-dimensions of financial access, depth, and efficiency, in conjunction with ecological footprint measurements related to consumption, exports, production, and imports. The findings established that overall FD and financial institution development, along with their sub-indices, exhibited an inverted U-shaped relationship with all types of environmental footprints considered by [49]. However, financial market development did not exhibit a nonlinear relationship with the environmental footprint as considered in the study. Though, Ashraf et al.'s [49] study also overlooked one significant aspect of FD, which is the financial stability of financial markets and institutions.

Importantly, the study of [50] established a U-shaped financial-environmental relationship in a prominent economy of China. Specifically, the study's findings confirmed a U-shaped relationship with both industrial solid waste and sulfur dioxide. However, Wang et al.'s [50] study lacked comprehensiveness in assessing financial development, as it solely relied on per capita deposits and loans for measuring FD. Additionally, [2,[51], [52], [53], [54], [55], [56], [57], [58]] also established FEKC in various study contexts. A recent study by [59] also examined an inverted U-shaped relationship between FD and CO_2_ in China. This study established that FD has a notable inverted U-shaped impact on CO_2_. However, [59] proxied FD using the total deposits and total loans to GDP ratio, which represents the depth of bank intermediation, but excluded other aspects of financial system development in China. It can be observed that a few studies, including those by [48,49,52,55] have made efforts to test the FEKC including Australia as one of the sample countries in their empirical analysis. Interestingly, these studies employed different panel time series techniques to enhance their study objective and establish FEKC within their study context. However, the existence of FEKC in the Australian economy remains unknown because these studies were unable to provide country-specific conclusions on the FEKC. More importantly, to the extent of the researchers' understanding, no empirical work has specifically pursued to test the FEKC in Australia as a single-country analysis. Additionally, most empirical works on FEKC have not comprehensively explored all aspects of FD, including financial depth, financial efficiency, financial access, and financial stability. There is a gap in in-depth research that considers the holistic nature of FD that tests the presence of the Kuznets curve. Therefore, this underscores the need for further investigation to test whether the Kuznets curve is applicable to the FD of the Australian economy.

## 3 Methodology

This section discusses the methodological strategy, model construction, and data that is adopted and used to address and enhance the objectives of this study.

### 3.1 Model construction

As mentioned earlier, this research utilizes the EKC framework to understand the FD-EQ nexus in Australia. To reach the aim of the study, we replace the traditional EKC framework, using EQ measurements instead of Sulfur Dioxide used in the traditional EKC framework, and FD instead of GDP per capita. Following the studies of [29,54,56,57], we use the following economic model, presented in Equation (1).

(1) $${EQ}_{t}=f\;\left({FD}_{t,}{FD}_{t,}^{2}{CV}_{t}\right)$$

Where, ${EQ}_{t}$ is the environmental quality, ${FD}_{t}$ is financial development, ${FD}_{t,}^{2}$ is the square of FD and ${CV}_{t}$ represents selected control variables of the model. To account for the presence of FEKC in Australia, we incorporated the FD^2^ into the economic model mentioned above. Thus, to capture the FEKC in Australia, the coefficient related to FD should be exhibited as positive and statistically significant, whereas the FD^2^ should be demonstrated as negative and statistically significant. An inverted U-shape exists when $\beta_{1}>0\;and\;\beta_{2}<0$ in Model 4. Additionally, to ascertain the turning point, calculate the partial derivative of Equation (6) with respect to the FD variable, as shown in Equation (2). Ultimately, by equating the partial derivatives to zero, the turning point can be calculated as given in Equation (3):

(2) $$\frac{\partial {EQ}_{t}}{{FD}_{t}}=\beta_{1}+2\beta_{2}{FD}_{t}$$

(3) $${FD}_{t}=\left[\frac{\beta_{1}}{2\beta_{2}}\right]$$

Additionally, based on the existing empirical works, we use economic growth, urbanization, energy use, foreign direct investments, and trade openness as control variables in the model.

The theoretical ground for including economic growth as a control variable is based on the EKC framework of [38], which explains an inverted U-shaped relationship with environmental degradation. Empirically, the EKC has been tested in various contexts and its presence confirmed [3,23,29,45,60,61]. In addition to the FEKC, present research tests the existence of EKC in the Australia and employs a square of economic growth to enhance it. Therefore, economic growth is anticipated to positively influence EQ, as it initially leads to increased environmental degradation [45]. Moreover, the expected impact of the GDP^2^ is negative because, when economic progress reaches a certain threshold level, environmental degradation drops down due to the transformation of the economic structure toward a service-dominated economy and the implementation of more sustainable technologies and practices to the economy [45].

Additionally, energy consumption is included as one of the control variables because higher energy consumption signifies increased reliance on natural gas and fossil fuels, leading to raised levels of pollution and resource degradation [62,63]. However, green technologies and innovations aid efficient energy utilization, thus aiding in the reduction of environmental harm [64], which is supposed to have either a positive or negative effect on EQ. Typically, urban growth habitually results in reducing air and water quality with diverse environmental changes such as solid waste, and global climate impacts [65]. However, in affluent cities, urbanization can yield positive environmental outcomes due to stringent environmental regulations, technological advancements, and innovative infrastructure developments, such as transportation and a shift toward service-oriented industries [65]. Consequently, urbanization is incorporated as a control variable in our model, as it is reasonable to anticipate either a negative or positive influence of urban growth on EQ in Australia.

The trade-environmental relationship is often discussed in terms of two possible ways: the impact of scale and the impact of composition. In the scale effect, trade facilitates economic advancement, leading to increased production and consumption, which, in turn, can negatively affect the environment [66]. However, the trade-environmental relationship tends to be favorable to the environment in rich countries due to the stringent environmental policies on trade [66]. Therefore, as a developed economy, we anticipate that trade will have a beneficial effect on EQ in Australia. Importantly, Foreign Direct Investments (FDI) is a vital mechanism for economic growth, but it also presents environmental pollution challenges to the host economy [67]. Additionally, FDI facilitates the transfer of innovative green technologies and sustainable practices that contribute to a healthier atmosphere by reducing GHG which is known as the pollution halo effect [67]. However, developed economies have the capacity to absorb the FDI, which enables them to enhance both EQ and productivity simultaneously [68]. As a developed economy, it is reasonable to anticipate a positive effect of FDI on EQ.

Equation (1) can be rewritten to incorporate the control variables within the broader economic framework as shown in Equation (4):

(4) $${EQ}_{t}=\alpha +{\beta_{1}FD}_{t}+{\beta_{2}{FD}^{2}}_{t}+\beta_{3}{GDP}_{t}+{\beta_{4}GDP}_{t}^{2}+\beta_{5}{ENG}_{t}+\beta_{6}{TO}_{t}+\beta_{7}{FDI}_{t}+\beta_{8}{URN}_{t}+\varepsilon_{t}$$

Where, ${GDP}_{t}$ and ${GDP}_{t}^{2}$ are per capita GDP and square of per capita GDP. ${\;ENG}_{t}$, ${TO}_{t}$, ${\;FDI}_{t}$, and ${URN}_{t}$ indicate energy consumption, trade openness, foreign direct investment, and urbanization respectively. From β_1_ to β_8_ estimate the coefficient of predictor variables. $\varepsilon_{t}$ denotes the model's residual term, and t presents the time variable. Additionally, to tackle the matter of exponential deviation in the data series, a log transformation is applied, resulting in the log model presented in Equation (5) below.

(5) $${lnEQ}_{t}=\alpha +{\beta_{1}lnFD}_{t}+{\beta_{2}{lnFD}^{2}}_{t}+\beta_{3}{lnGDP}_{t}+{\beta_{4}lnGDP}_{t}^{2}+\beta_{5}\;\ln\;{ENG}_{t}+\beta_{6}{lnTO}_{t}+\beta_{7}\;\ln\;{FDI}_{t}+\beta_{8}{lnURN}_{t}+\varepsilon_{t}$$

More importantly, the enforcement of environmental policies, rules, and regulations has exerted a substantial influence on environmental quality and serves as an effective tool for controlling environmental pollution of the economy, while encouraging the adoption of environmentally friendly technologies and production processes [69,70]. Specifically, the existence of robust environmental policies, rules, and regulations is basic for enhancing the EQ targets expected by governing bodies [70]. However, implementing unfitting environmental regulations such as inappropriate carbon taxes, does not contribute to the achievement of an economy's environmental targets. Instead, such regulations may induce firms to increase their current levels of extraction, leading to greater pollution [70,71]. Furthermore, the enforcement of environmental policies, rules and regulations can become an additional financial burden on firms, requiring them to allocate more financial resources for expenditures related to pollution management function [70]. This study incorporates a dummy variable to estimate the impact of environmental policies, rules, and regulations. Specifically, carbon tax is chosen as the measure of environmental policies, rules, and regulations. In Australia, the carbon tax was introduced in 2011 and effectively implemented from July 1, 2012. However, in 2014, a carbon tax was rebated by the government. We employ a binary dummy variable, denoted as ‘0′ for the pre-carbon tax period spanning from 1980 to 2011 and 2015–2021, and ‘1′ for the time frame starting from 2012 to 2014, representing the time period following the implementation of the carbon tax in Australia. It is anticipated carbon tax will reduce GHG in Australia.

Equation (5) is rearranged as follows (equation (6)) by incorporating the dummy variable $\left(D_{t}\right)$ into the model.

(6) $${lnEQ}_{t}=\alpha +{\beta_{1}lnFD}_{t}+{\beta_{2}{lnFD}^{2}}_{t}+\beta_{3}{lnGDP}_{t}+{\beta_{4}lnGDP}_{t}^{2}+\beta_{5}\;\ln\;{ENG}_{t}+\beta_{6}{lnTO}_{t}+\beta_{7}\;\ln\;{FDI}_{t}+\beta_{8}{lnURN}_{t}+\vartheta D_{t}+\varepsilon_{t}$$

### 3.2 Methodological strategy

At the outset, we perform a stationary test to determine whether the dataset is stationary, as this is a prerequisite for time series analysis. In this empirical work, we worked with the Augmented Dickey-Fuller test to check for the unit root in the dataset. Mainly, we employ the Autoregressive Distributed Lag (ARDL) bound test to empirically explore the presence of the FEKC in the Australian context. [47] also employed a similar estimation strategy to estimate the FEKC. The ARDL model is suitable for researchers as it can be applied whether the individual regressor's order of integration is I (0) or I (1) [72]. In addition, the ARDL procedure is well-suited for small samples, and it estimates a dynamic error correction model through a straightforward linear transformation. Moreover, the ARDL estimation technique has the dual capacity to estimate both long-run and short-run dynamics, which is crucial for grasping the immediate and long-term impacts of FD on EQ, thereby validating the FEKC. The ARDL model, as estimated, is shown in Equation (7), with the optimal lags for the cointegration equation determined based on the Akaike Information Criterion.

(7) $$\Delta {lnEQ}_{t}=\beta_{0}+\beta_{1}{lnEQ}_{t-1}+\beta_{2}{lnFD}_{t-1}+\beta_{3}{lnFD}_{t-1}^{2}+\beta_{4}{lnGDP}_{t-1}+\beta_{5}{lnGDP}_{t-1}^{2}+\beta_{6}{lnENG}_{t-1}+\beta_{7}{lnTO}_{t-1}+\beta_{8}{lnFDI}_{t-1}+\beta_{9}{lnURN}_{t-1}+\beta_{10}D_{t}+\sum_{i=1}^{p}\delta_{1}{\Delta lnEQ}_{t-i}+\sum_{i=1}^{p}\delta_{2}{\Delta lnFD}_{t-1}+\sum_{i=1}^{p}\delta_{3}{\Delta lnFD}_{t-1}^{2}\sum_{i=1}^{p}\delta_{4}{\Delta lnGDP}_{t-1}+\sum_{i=1}^{p}\delta_{5}{\Delta {lnGDP}^{2}}_{t-1}+\sum_{i=1}^{p}\delta_{6}{\Delta lnENG}_{t-1}+\sum_{i=1}^{p}\delta_{7}{\Delta lnTO}_{t-1}+\sum_{i=1}^{p}\delta_{8}{\Delta lnFDI}_{t-1}+\sum_{i=1}^{p}\delta_{9}{lnURN}_{t-1}+\varepsilon_{t}$$

Following that, cointegration among the variables under investigation is assessed through bound testing, as detecting a long-term relationship between them enables the analysis of long-run coefficients. Subsequently, Long-run coefficients are tested. Next, the Error Correction Model (ECM) is used to capture how the model adjusts towards equilibrium, with the ECM model outlined in Equation (8).

(8) $$\Delta \ln\;{EQ}_{t}=\delta_{0}+\sum_{i=1}^{p}\delta_{1}{lnEQ}_{t-1}+\sum_{i=0}^{p}\delta_{2}{\Delta lnFD}_{t-i}+\sum_{i=0}^{p}\delta_{3}{\Delta {lnFD}^{2}}_{t-i}+\sum_{i=0}^{p}\delta_{4}\Delta {lnGDP}_{t-i}+\sum_{i=0}^{p}\delta_{5}{\Delta {lnGDP}^{2}}_{t-i}+\sum_{i=0}^{p}\delta_{6}{\Delta lnENG}_{t-i}+\sum_{i=0}^{p}\delta_{7}\Delta {lnTO}_{t-i}+\sum_{i=0}^{p}\delta_{8}{\Delta lnFDI}_{t-i}+\sum_{i=1}^{p}\delta_{9}{lnURN}_{t-i}+\delta_{10}D_{t}+{\psi {ECT}_{t-1}+\varepsilon }_{t}$$

Cointegration between EQ and the predictors implies that there may be at least one-directional causality between these variables. Therefore, this study conducted a Granger causality test, following [73], to examine causality that is not implied by the ARDL bound test. Equations (9), (10)) outline the causality models that Y does not Granger-cause X and X does not Granger-cause Y.

(9) $$Y_{t}=\varsigma_{0}+\varrho_{1}Y_{t-1}{+}_{\ldots \ldots \ldots ..}+\varrho_{k}Y_{t-k}+\varepsilon_{1}X_{t-1}{+}_{\ldots \ldots .}\varepsilon_{k}X_{t-k}+\omega_{t}$$

(10) $$X_{t}=\zeta_{0}+\vartheta_{1}X_{t-1}{+}_{\ldots \ldots \ldots ..}+\vartheta_{k}X_{t-k}+\xi_{1}Y_{t-1}{+}_{\ldots \ldots .}\xi_{k}Y+\varphi_{t}$$

### 3.3 Variables and data

This study spans from 1980 to 2021, based on the availability of data, with most of the information being in annual intervals; this research is confined to annual data. The property of all pertinent data is time series and gathered from several secondary sources including the World Bank database, IMF database, and Bloomberg database. Table 1 below outlines the dimensions employed to measure each variable.

**Table 1:** Summary of variables, proxies utilized in the study.

Variable	Proxy Variable
EQ (Environmental Quality)	Total greenhouse gas emissions (Metric tons)
FD (Financial Development)	Overall financial development index
	Stock price volatility (%)
	Bank credit to bank deposits (%)
GDP (Economic Growth)	Gross domestic production per capita (Current US$)
ENG (Energy Consumption)	Primary energy consumption per capita (kWh/person)
FDI (Foreign Direct Investment)	Net inflows of direct investment (Balance of Payments, current US$)
TO (trade Openness)	Exports and imports of goods and services as a percentage of GDP
URB (Urbanization)	Urban population as a percentage of the total population

More importantly, in this study, FD is assessed using three proxies. In this context, we use the overall FD index provided by the IMF, the ratio of bank credit to bank deposits (%), and stock price volatility. The IMF's FD index encompasses the development of financial markets and financial institutions, including aspects of financial access, financial depth, and efficiency. Consequently, we employ rest of the proxies to quantify the financial stability of financial institutions and financial markets, respectively.

**Fig. 1:**
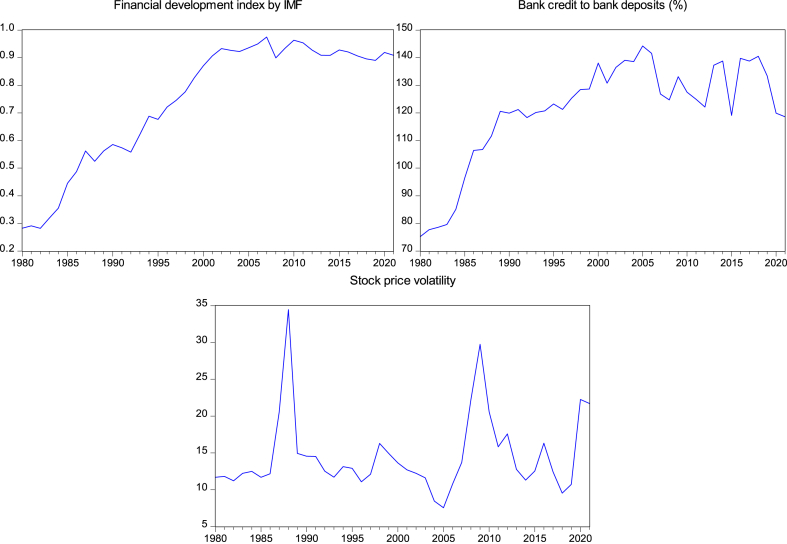
Time series plots of FD proxies.

**Fig. 2:**
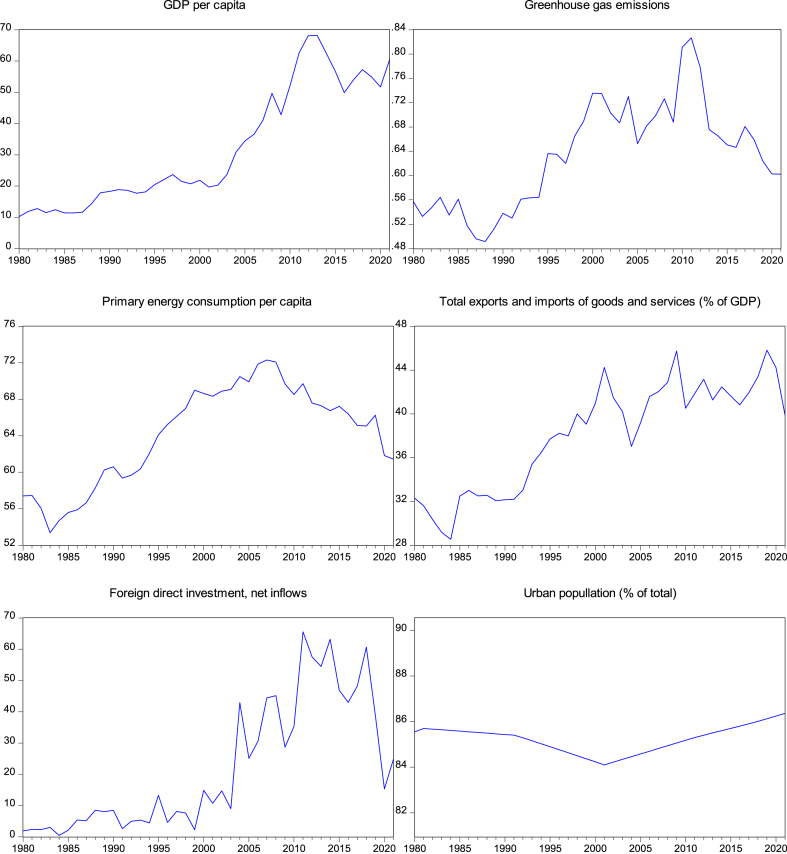
Time series plots of GHG, economic growth, energy consumption, trade, FDI and urbanization.

Fig. 1 displays the graphical representations of the time series plots that employed to gauge FD. Additionally, Fig. 2 graphically presents the time series plots of the proxies for EQ and other control variables utilized in this study.

## 4 Results from empirical analysis and discussion

Table 2 summarizes the descriptive statistics for the studied variables used in this analysis. All the variables exhibited moderate skewness. GDP per capita and EQ exhibited right-skewness, while the remaining variables exhibited left-skewness. Additionally, the probability values from the Jarque-Bera test indicate that all the variables conform to a normal distribution.

**Table 2:** Descriptive statistics.

**Description**	**LnEQ**	**LnFD**	**LnGDP**	**LnENG**	**LnTO**	**LnFDI**	**LnURB**
Mean	20.256	−0.652	10.192	11.065	3.631	23.203	4.445
Median	20.278	−0.357	9.995	11.092	3.676	23.201	4.447
Maximum	20.532	0.358	11.129	11.188	3.824	24.906	4.458
Minimum	20.012	−3.245	9.230	10.884	3.351	19.875	4.432
Kurtosis	2.1084	3.884	1.604	1.962	1.959	2.388	2.210
Skewness	0.0010	−0.613	0.100	−0.458	−0.487	−0.401	−0.243
Jarque-Bera	1.390	2.353	3.479	3.351	3.557	1.781	1.505
Probability	0.498	0.436	0.175	0.187	0.168	0.410	0.470
Std. Dev.	0.1359	1.031	0.616	0.086	0.133	1.255	0.006
Sum	850.778	−27.398	428.092	464.738	152.506	974.538	186.706
Sum Sq. Dev.	0.758	43.602	15.564	0.306	0.726	64.656	0.001
Observations	42	42	42	42	42	42	42

Before proceeding with the ARDL model, it is required to verify the level of integration of the data set. In this analysis, we utilized the Augmented Dickey-Fuller (ADF) test to determine the stationarity of the dataset studied in this study. Here, we test the null hypothesis *H*_0_: *β* = 0 against the alternative hypothesis *H*_1_: *β* < 0. Table 3 provides a summary of the results from the ADF test. It is evident that none of the variables are stationary at level series, except LnFD and LnFD^2^. However, all the data confirm level of integration at the first difference, and it permits to use ARDL model in study variables for enhancing the objectives.

**Table 3:** Stationarity test results.

**Variable**	**Level series**	**1**st **difference**
LnEQ	−1.487	−6.586∗∗∗
LnFD	−2.731∗	−7.420∗∗∗
LnFD^2^	−3.708∗∗	−5.556∗∗∗
LnGDP	−0.870	−4.990∗∗∗
LnGDP^2^	−0.776	−4.968∗∗∗
LnENG	−1.229	−5.197∗∗∗
LnTO	−1.455	−5.982∗∗∗
LnFDI	−1.611	−9.987∗∗∗
LnURB	−1.667	−7.215∗∗∗

Calculations by the authors.

Note: ∗∗∗, ∗∗ & ∗ denote significance at 1 %, 5 %, and 10 % level, respectively.

Next, we estimated the ARDL model and assessed the cointegration between all studied variables to derive the long-term and short-term effects. The Akaike Information Criteria was applied to select the optimal lag length, and it confirmed that optimal lag length is 1, 0, 0, 1, 0, 1, 0, 1, 1, 0 for LnEQ, LnFD, LnFD^2^, LnGDP, LnGDP^2^, LnENG, LnTO, LnFDI, and LnURB. The stastistcal estimationsof the bound test is expressed in Table 4. It confirms cointegration among the study's variables, signifying the presence of a long-run equilibrium relationship between the LnEQ, LnFD, LnFD^2^, LnGDP, LNGDP^2^, LnENG, LnTO, LnFDI, and LnURB. Furthermore, the verification of a long-run relationship is an essential condition for testing the long-run coefficients, which are given in Table 5. Importantly, 97 % of R^2^ value suggests that EQ are comprehensively explained by all the repressors studied in the long run model of the analysis. Moreover, the long-run fitted model is valid since the Durbin-Watson statistic is higher than the R^2^ value.

**Table 4:** Bound test results.

F-statistic	7.7255∗∗∗	Critical Values	I (0)	I (1)
		10 %	1.76	2.77
		5 %	1.98	3.04
		1 %	2.41	3.61

Note: ∗∗∗ denotes significance at the 1 % level.

**Table 5:** Long-run coefficient estimates.

**Variable**	**Coefficient**	**Std. error**	**t-statistic**	**Prob.**
LnFD	0.0444	0.0213	2.0853	0.0474∗∗
LnFD^2^	−0.4852	0.1495	−3.2446	0.0033∗∗∗
LnGDP	2.7918	0.9757	2.8611	0.0084∗∗∗
LnGDP^2^	−0.1401	0.0465	−3.0105	0.0059∗∗∗
LnENG	1.0029	0.5856	1.7126	0.0992∗
LnTO	0.0062	0.0046	1.3401	0.1923
LnFDI	−0.0339	0.0170	−1.9984	0.0567∗
LnURB	3.0866	5.9090	0.5223	0.6060
Dummy	−0.2012	0.0976	−2.0608	0.6508
C	9.3061	30.6952	0.3031	0.7643

Durbin-Watson statistic: 1.5802, R-squared: 0.9710, Adjusted R-squared: 0.9537.

Note: ∗∗∗, ∗∗ & ∗ denote significance at the 1 %, 5 %, and 10 % levels, respectively.

**Fig. 3:**
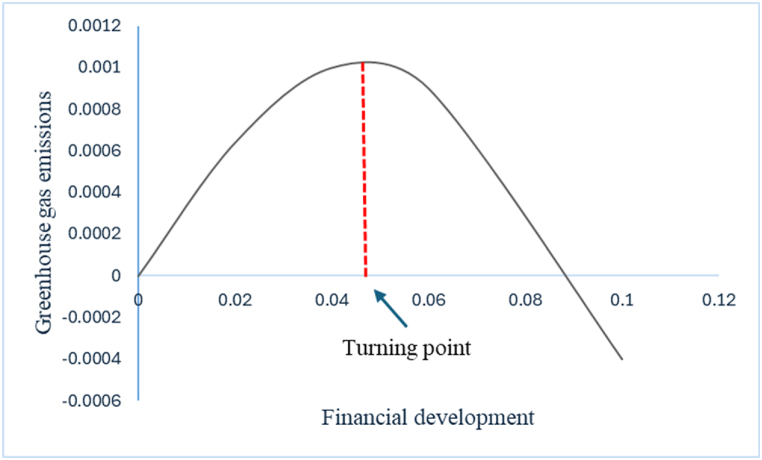
Financial environmental Kuznets curve (FEKC).

Basically, In long run, LnFD, LnGDP, LnFD^2^, LnGDP^2^, LnFDI, LnENG, and carbon tax exhibit statistical significance. FD positively affects GHG, indicating that in Australia, increased FD contributes to worsening EQ by producing more GHG. The estimated coefficient indicates that a 1 % change in Australia's financial system results in a 0.04 % change in emissions. It is clear that the financial system in Australia still prioritizes profitability over environmental sustainability, channeling financial resources towards profitable ventures rather than environmentally sustainable ones. This finding of FD is consistent with the discoveries of [2,45,47,48,50,57]. According to the empirical model presented in this work, the analysis results verify a negative and statistically significant impact of the square of FD on GHG, implying an enhancement in EQ. Furthermore, it establishes an inverted U-shaped relationship between FD and EQ in Australia, proving the existence of the FEKC. The coefficient value is significant, demonstrating that a 1 % change in the FD^2^ directs to a 0.48 % change in GHG. The presence of FEKC in Australia suggests that the country's financial system has shifted its focus toward sustainable financing, which aims to achieve long-term environmental sustainability. Similarly, the FEKC hypothesis has been substantiated by studies conducted in various economic contexts, as exemplified by [2,[44], [45], [46], [47],^50^]. Importantly, the turning point is 0.0458, which emphasizes that in the early phase of FD, it degrades the environment. However, it turns positively towards EQ after FD reaches the 0.0458 level, thereby improving environmental conditions. Refer to Fig. 3 for a graphical presentation of the FEKC.

Among other significant findings, it is evident that economic growth is contributing to a decline in EQ, generating 2.8 % of emissions for every 1 % increase in the Australian economy. However, the LnGDP^2^ exhibits a statistically significant negative coefficient which confirms the EKC in the Australia. Several factors may contribute to the presence of the EKC in the Australian context, such as the transition from an industrialized economy to a service-oriented economy, technological advancements like energy efficiency and green technologies, and policy interventions and global integration towards sustainability. Moreover, this finding aligns with the validation of the EKC as previously demonstrated by the studies of [3,23,60,61] in different economies. Additionally, this finding supports the empirical findings of [33,74] within the Australian context.

It is noteworthy that primary energy consumption significantly contributes to mounted emissions in Australia, as a 1 % growth in energy utilization corresponds to a 1 % expand in toxic emissions. This positive effect can be traced back to the high consumption of fossil energy sources. Additionally, it is worth mentioning that Australia ranks as the world's 10^th^ largest coal consumer. This empirical evidence supports the existing pieces of evidence of [75,76] in the Australian context. Interestingly, FDI (FDI) reported a statistically significant and negative effect on GHG. This implies that FDI causes to an advancement in EQ in Australia by lowering toxic emissions. Additionally, the adverse influence of FDI on GHG supports to confirmation of the pollution halo effect within the Australian economy. The presence of pollution halo effect indicates that the Australian economy actively attracts FDIs that transfer green technologies and practices, ultimately enhancing EQ. This result aligns with the empirical findings reported by [77].

Moreover, environmental regulation has a negative effect, and it is statistically insignificant, indicating that Australia's existing carbon tax scheme does not effectively impact to decrease GHG level. In contrast, the findings of [78,79] support the knowledge that a carbon tax can successfully reduce emissions in Australia and this empirical finding also challenges the existing evidence. Finally, the long run influence of urbanization on EQ in Australia is insignificant within the Australian context. However, this finding challenges the results of previous studies by [29,80,81]. Besides, by challenging the observed outcomes of [21,82] this research affirms the insignificance role of trade openness in determining EQ in Australia.

The F-statistic from the short-run bound test is 6.8642, surpassing the upper limits, which confirms the existence of short-run cointegration among the analyzed variables and allows for the assessment of short-run estimates. The estimation results of short-run coefficient are shown in Table 6. Additionally, the ECT is negative and statistically significant, indicating that EQ returns to its equilibrium at a rate of 45.23 % after variations in all predictor variables.

**Table 6:** Short-run coefficient estimates.

Variable	Coefficient	Std. error	t-statistic	Prob.
D (LnFD)	0.0156	0.0151	1.0323	0.3126
D (LnFD^2^)	−0.0299	0.0068	−4.3606	0.1528
D (LnGDP)	0.0055	0.0056	0.9962	0.3287
D (LnGDP^2^)	0.3469	0.1869	1.8562	0.2752
D (LnENG)	0.3436	0.2049	1.6767	0.0071∗∗∗
D (LnTO)	−0.5065	0.0928	−5.4580	0.0000∗∗∗
D (LnFDI)	0.0164	0.0056	2.8844	0.0084∗∗∗
D (LnURB)	−40.3000	4.3473	−9.2700	0.0000∗∗∗
D (Dummy)	−0.0059	0.0273	−0.2170	0.8301
CointEq (-1)∗	−0.4523	0.0434	−10.408	0.0000∗∗∗

R-squared: 0.8415, Adjusted R-squared: 0.8239, Durbin-Watson statistic: 1.5802.

More importantly, the FEKC does not appear in the short run in Australia, which contradicts the findings of [46,47]. Additionally, the EKC also does not seem to valid in the short run. Among the other variables studied, LnENG, LnTO, LnFDI, and LnURB are statistically significant. In the immediate term, FDI and energy usage elevate GHG levels, thereby worsening EQ. Specifically, in the long run, FDI appears to foster the creation of a pollution halo effect, but pollution halo effect is not observed in Australia in the short term. The variables urbanization and trade openness, which play a neutral role in the long run, play a vital role in improving EQ by lowering toxic emissions in the short run. Pressure on trade standards and regulations, technological infrastructure development in urban areas, and technological advancements may contribute to a positive effect on EQ from urbanization and trade openness in the short run. Notably, the role of carbon tax in determining the degree of EQ is insignificant in the short run. This may be due to other significant economic proxies such as economic growth, offsetting the impact of the carbon tax in the short run. Furthermore, the fitted short run model is not a spurious model, and it well explains 84.39 % of the variation in EQ by the explanatory variables included in the empirical model.

Next, the econometric output of the causality test, which was employed to the first-difference data, are summarized in Table 7. It can be observed that LnFD, LnFD^2^, LnGDP, LnGDP^2^, LnENG, and LnFDI indicate unidirectional causality leading to EQ, further strengthening the evidence of cointegration in this study. However, no causality has been reported between trade openness and EQ, as well as between urbanization and EQ.

**Table 7:** Granger Causality test results.

Null Hypothesis	F- statistic	Decision
LnFD ↛ LnEQ	0.5501∗∗ [0.0256]	One-way causality from LnFD to LnEQ
LnEQ ↛ LnFD	0.1721 [0.8425]	
LnFD^2^^↛^ LnEQ	1.0457∗∗ [0.0362]	One-way causality from LnFD^2^ to LnEQ
LnEQ ↛ LnFD^2^	0.0544 [0.9471]	
LnGDP ↛ LnEQ	0.1852∗ [0.0831]	One-way causality from LnGDP to LnEQ
LnEQ ↛ LnGDP	0.2148 [0.8077]	
LnGDP^2^ ↛ LnEQ	0.2869∗ [0.0523]	One-way causality from LnGDP^2^ to LnEQ
LnEQ ↛ LnGDP^2^	0.2609 [0.7718]	
LnENG ↛ LnEQ	5.4789∗∗∗ [0.0085]	One-way causality from LnENG to LnEQ
LnEQ ↛ LnENG	0.0918 [0.9125]	
LnTO ↛ LnEQ	2.8170 [1.0734]	No causality
LnEQ ↛ LnTO	1.2006 [0.3131]	
LnFDI ↛ LnEQ	3.2130∗ [0.0523]	One-way causality from LnFDI to LnEQ
LnEQ ↛ LnFDI	1.7968 [0.1808]	
LnURB ↛ LnEQ	1.9838 [0.1527]	No causality
LnEQ ↛ LnURB	3.1991 [0.1530]	

Note: ∗∗∗, ∗∗ & ∗ denote significance at 1 %, 5 %, and 10 % level, respectively.

**Fig. 4:**
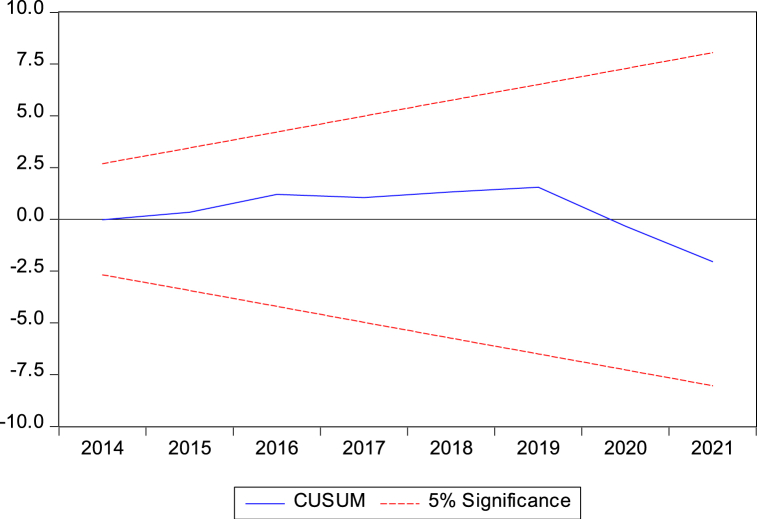
Plots of CUSUM Test.

**Fig. 5:**
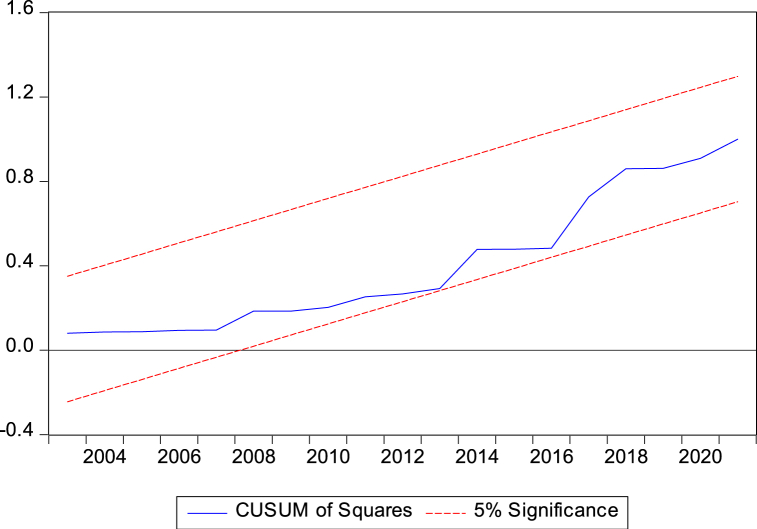
Plots of CUSUM of Squares Test.

Finally, to evaluate the robustness of the fitted ARDL model, several diagnostic tests were performed. The outcomes of the heteroskedasticity test, correlation LM test, and Jarque-Bera statistics are shown in Table 8 (refer appendix). Accordingly, these tests confirm the non-existence of serial correlation in the residuals, indicating homoscedasticity of residuals, and that residuals follow a normal distribution. Finally, the CUSUM test and CUSUM of Square test results, as shown in Figs 4 and 5 (refer appendix), further confirm the stability of the selected time series analysis in both the long run and short run, as the statistics lie within the 5 % confidence bounds.

## 5 Conclusion and policy implications

EQ is a vital aspect of the modern world, and economic leaders are continuously committed to enhancing it. In this regard, FD in the economy plays a substantial role in improving EQ by guiding financial markets and financial institutions. This scholarly work aims on examining the link between FD and EQ in the Australian economy to bridge existing knowledge gaps in the research domain. Specifically, this research seeks to grasp the linkage between FD and EQ, which is recognized as the FEKC, by adopting the theoretical framework of the EKC. This study is unique compared to existing wisdom in this research domain because, to address the research gap, It takes into account all aspects of FD, such as financial depth, access, efficiency, and the stability of financial markets and institutions. The study covers the time frame from 1980 to 2021 based on the data availability.

The main findings are outlined below: First, a long-term association among studied variables was found. In the long run, FD was observed an adverse effect on EQ. Additionally, the square term of FD exhibited a favorable impact on EQ, confirming an inverted U-shaped relationship between FD and EQ (known as the FEKC) in Australia. However, short-term results prove the non-existence of the FEKC. Moreover, the study established the EKC in the long run and reaffirmed similar results as the FEKC in the short run. Furthermore, energy consumption was found to negatively impact EQ. FDI validated the pollution halo effect in the long run while worsening EQ in the short run. Notably, the study found that the carbon tax played a neutral role in changing EQ in both the long run and the short run. Finally, in short run, trade openness and urbanization were shown to positively impact the improvement of the EQ of the Australian economy.

According to the empirical results, several policy suggestions can be provided for policymakers in the Australian economy. Firstly, Australia's financial system should be strengthened to reduce present environmental pollution by channeling financial resources into sustainable investment avenues, such as green production and renewable energy generation. In this context, both financial markets and financial institutions should be reinforced to direct monetary resources toward investments that address environmental challenges. Regular monitoring of the financial flows within the financial system is essential. In addition, policymakers should focus on to the profit maximization objectives of financial intermediaries and participants in the financial system, especially since FD has a negative impact in its initial stages. Additionally, fostering environmental awareness among financial intermediaries, and investors shareholders, could help achieve the environmental targets set by the Australian government and benefit the entire community in the country. Importantly, policy implications should primarily be adaptive and dynamic to the different stages of FD in Australia. Mainly, policy implications should be more adaptive and dynamic to the different stages of the FD in Australia.

Furthermore, policymakers should encourage highly polluting industries to implement environmentally friendly production processes, adopt innovative energy-efficient technologies, and allocate resources to renewable energy initiatives. Specifically, increasing the supply of renewable energy with providing monetary support to individuals and industries to encourage the adoption of renewable energy sources, ultimately reducing pollution. Policymakers should also welcome ecologically friendly FDIs into the country and strengthen policies and regulations to mitigate their adverse effect in the short run. Regarding carbon tax, the study suggests that it does not significantly affect EQ in Australia. Therefore, policymakers should consider implementing and monitoring effective environmental regulations as a solid way forward without altering policy decisions or allowing subsidies to high-polluting industries to maintain political interests. Lastly, the key findings of this scholarly work can diliver as a guideline for other developed nations to regulate their financial systems to enhance EQ.

### 5.1 Limitations and future research direction

This research gives valuable insights to validate the FEKC in Australia. But, it is necessary to emphasize that these results cannot be completely generalized to all advanced countries for policy implications due to different economic and financial settings. Therefore, comparative studies analyzing the validity of the FEKC in several advanced economies with diverse economic and financial contexts are essential. In this study, we were unable to include the ecological footprint, which evaluates human impact on the environment. Therefore, it is imperative to urge future researchers to incorporate the ecological footprint in order to assess EQ within the study context. Additionally, due to data limitations, we utilized two proxy variables to gauge the stability of the financial market and financial institutions. However, the proxies we employed cover the stability of stock markets and banking institutions only and exclude the stability of other markets, such as the insurance market. This emphasizes a need for future researchers to consider broader measures for capturing the financial stability of financial markets and institutions.

## Data availability statement

The complete structured data set used for the study may be supplied by the corresponding author on reasonable request.

## CRediT authorship contribution statement

**Ambepitiya Wijethunga Gamage Champa Nilanthi Wijethunga:** Writing – review & editing, Writing – original draft, Validation, Supervision, Software, Methodology, Investigation, Formal analysis, Data curation, Conceptualization. **Mohammad Mafizur Rahman:** Writing – review & editing, Supervision, Conceptualization. **Tapan Sarker:** Writing – review & editing, Supervision.

## Declaration of competing interest

The authors declare that they have no known competing financial interests or personal relationships that could have appeared to influence the work reported in this paper.

## Figures and Tables

**Tabile 8:** The results of diagnostic tests

Breusch-Godfrey Serial Correlation LM Test	0.556699 [0.5806]
Heteroskedasticity Test: Breusch-Pagan-Godfrey	0.313077 [0.9716]
Jarque-Bera	1.711461 [0.4249]

Parenthesis “[.]” indicates the probability values.

## Abbreviations

FD: Financial Development
EQ: Environmental Quality
FEKC: Financial Environmental Kuznets Curve
EKC: Environmental Kuznets Curve
GHG: Greenhouse gas emissions
CO2: Carbon emissions
ARDL: Autoregressive Distributed Lag Model

## Footnotes

1 Source: Reserve Bank of Australia (RBA), 2023.

2 Source: Commonwealth Scientific & Industrial Research Organization (CSIRO), 2023.
